# Alone and together: registered nurses’ experiences of work satisfaction in municipal home healthcare

**DOI:** 10.1186/s12912-024-02051-3

**Published:** 2024-06-05

**Authors:** Therese Stien, Karin Josefsson

**Affiliations:** 1 Faculty for Nursing and Health Science, NORD University, Bodø, 8026 Norway; 2 Department of Health Sciences, Faculty of Health, Science, and Technology, Karlstad University, Karlstad, 651 88 Sweden

**Keywords:** Work satisfaction, Municipal home healthcare, Registered nurse, Interview

## Abstract

**Background:**

The need for advanced home healthcare (HHC) is expected to increase, with registered nurses (RNs) as key figures. Given the difficulties recruiting and retaining RNs in the HHC sector, understanding their work satisfaction is imperative.

**Aim:**

This study aimed to explore RNs’ experiences of work satisfaction in the municipal HHC.

**Methods:**

Individual interviews were conducted with RNs (*n* = 8) in four municipalities in Norway. The data were evaluated using qualitative content analysis.

**Results:**

Work satisfaction in HHC was organised into one theme ‘alone and together’ under four categories—the patient, the co-worker, the registered nurse, and the organisation—and 15 subcategories, including patient diversity, supportive co-workers and professional environment, appropriate workload and responsibilities, and provision of preconditions for self-management.

**Conclusions:**

Patients, co-workers, and organisations were identified as crucial areas affecting RNs’ work satisfaction in the municipal HHC. Awareness of these areas is essential to promote RNs’ work satisfaction. Patients’ diversity adds positively to RNs’ work satisfaction. Notably, RNs working alone can affect their work satisfaction not only negatively but also positively.

**Supplementary Information:**

The online version contains supplementary material available at 10.1186/s12912-024-02051-3.

## Background

Norwegian home healthcare (HHC) is a publicly funded healthcare municipal service and provides care for everyone in need in the home [1]. In Norway, the majority of people who require HHC are older adults; HHC is the healthcare area with the highest expansion rate [2], exhibiting an increase of 11% from 2017 to 2021 [3]. The transferring of responsibility for patients from hospital-based care to HHC was politically motivated through the healthcare reform The Coordination Reform [4]. Norwegian public health has been increasingly focusing on healthy ageing and disease prevention; as the population ages, so does the number of people receiving HHC [5]. Furthermore, even though the risk of illnesses such as dementia has been decreasing [5], the number of people with dementia is increasing with an increase in the number of older adults.

Registered nurses (RNs), certified nursing assistants and nursing assistants with or without formal healthcare training provides a wide range of services and healthcare in municipal HCC [6]. The patients in HHC typically have progressive disease and varied and complex healthcare needs and are cared for by RNs, who have diverse responsibilities and work tasks, including a vital assessment of the patient’s health needs and providing services related to personal hygiene, wound care, and dialysis treatment. There are indications that HHC is being colonised by a more medical ideology where urgent treatment and care are taking precedence over more slowly progressing treatment and care [6]. Older adults feel more secure when receiving healthcare in their own home environment [7]. Although RNs are necessary to meet the multifaceted needs of HHC, there is a systematic shortage of RNs, which negatively affects the quality of care received by patients [8].

Approximately half of the RNs in HHC wish to leave their jobs [9], and 20% of Norwegian RNs are no longer employed in healthcare 10 years after completing their nursing education [10]. The intent to remain at work is linked to work satisfaction [11, 12]. Among healthcare workers, work satisfaction is intricately associated with interpersonal relationships and interactions, including with patients, colleagues, and managers; work-related factors, such as the work itself, workload, working conditions, and opportunities related to personal development and career; experience of control and autonomy; job security; and leadership style [13]. These factors also influence RNs’ work satisfaction [13–16], which in turn is known to be associated with health [17, 18]. Healthcare professionals’ work satisfaction positively influences the quality of care provided to patients [19, 20]. Among HHC personnel, work satisfaction is shown to be moderate [21], which can be considered conspicuously high compared with how many who express wishes to leave the work [9], and also when the negative factors influencing the work environment are taken into consideration; for example, the Norwegian HHC personnel have the highest rate of sick leave in the country [22]. Thus, this study aimed to explore RNs’ experiences of work satisfaction in municipal HCC.

### Research question

How do registered nurses experience work when they are satisfied in municipal home health care?

## Methods

### Design

An explorative and inductive design was used [23]. Individual interviews with eight RNs were conducted from January to March 2023 in four municipalities in Norway. Data were analysed using the qualitative content analysis method [24–26]. The study is registered with number 866,828 in SIKT (the Norwegian Agency for Shared Services in Education and Research, which provides guidelines for insured privacy in research).

### Participants and procedure

The inclusion criteria were RNs employed in municipal HHC in 2023. The managers of HHC in 10 municipalities were informed by email about the study and were given an information letter, which they were asked to distribute to RNs in their employment. The information letter contained information about the performance of the interviews, ensuring confidentiality, how data are used and stored, and the researcher’s contact information in case of consent withdrawal. The RNs who wanted to participate contacted the first author by phone or email and provided informed consent. Before the interviews, oral consent was obtained. Three RNs withdrew their consent prior to the interviews, and one did not appear for the interview. Therefore, four RNs employed at HHC contacted and relayed the information letter to their colleagues, who contacted the first author. All RNs were guaranteed confidentiality and anonymous presentation of the results. Table 1 summarises the participants’ characteristics.

**Table 1:** Characteristics of the participants (*n* = 8)

Age	27–64 years (mean: 41 years, SD: 37 years)
Female	*n* = 6
Male	*n* = 2
Work experience municipal home healthcare	0.5–24 years (mean: 7 years, SD: 23.5 years)
Numbers worked in a rural area	*n* = 6
Numbers worked in a city area	*n* = 2

### Data collection

An interview guide (see Supplementary file) was developed for this study within the research team, which was discussed at seminars. To test the logistics, relevance, and clarity of the questions, one RNs with experience in HCC was interviewed. This interview did not lead to any changes in the guide and was therefore included in the data collection. Two main questions were used: (1) Can you tell me what it is like at work when you are most satisfied? and (2) Is there something that contributes to your work satisfaction that you wish to highlight? Follow-up questions were used when necessary—for example, in what way? Can you tell me more? The interview guide had a closing question: Is there anything you want to add before ending the interview? The interviews were audio-recorded and lasted for an average of 30 min (min - max 25–60 min).

### Data analyses

The data were analysed using qualitative content analysis [24–26]. Qualitative content analysis has an inductive approach; in other words, the categories are not determined in advance but emerge through the analysis. The interviews were transcribed. The analysis started by repeatedly reading the data in their entirety to obtain an overview of the content related to the study aim. Variations and similarities were identified, and statements related to the content area were divided into meaningful units. Thereafter, the analysis progressed from meaning units through condensed meaning units which were abstracted and labelled with codes, which were sorted into subcategories and then into main categories—in other words, an abstraction and interpretation of the text while preserving the core - and finally, an interpretation was done on one theme. The various stages were discussed between the researchers and in seminars several times. Table 2 provides an example of the data analysis.

**Table 2:** Example of the qualitative content analysis process

Meaning unit	Condensed meaning units	Code	Subcategory	Category	Theme
…and then there is a sense of freedom, that I can manage my day myself…	Managing the day myself gives a sense of freedom	*S*elf-manage gives a sense of freedom	To self-manage	The registered nurse	Alone and together

### Preunderstanding

The researcher’s experience of own work satisfaction as an RN in HHC did not lead to an understanding of the empirical data, preparation of the interview guide, or data analysis. To ensure an unbiased perspective, the researchers bracketed preconceived notions and had continuous reflections on how preunderstanding could influence the analysis. All the analysis steps were discussed in-depth under several seminars within the research team to ensure that the researchers’ preunderstanding did not take precedence over the data.

### Ethical consideration

The RNs’ personal information had to be respected by the researchers. The researchers strove to prevent discomfort and violation of integrity. All potential participants were given an autonomous choice to participate or not. It was made clear that the investigators did not work at the request of any of the RNs’ employers. There was no dependence between the participants and the researcher, which could have influenced the participants. The participants were informed that the audio-recorded interviews would only be heard by the researchers, that their identities would be protected, and that collected data would be acquired with no connections between name and work unit. Information about the possibility of being informed of the results was provided.

## Results

RNs’ experience of work satisfaction in municipal HHC was organised under the theme ‘alone and together’ with four categories—the patient, the co-worker, the registered nurse and the organisation—and 15 subcategories (Table 3).

**Table 3:** Registered nurses’ experiences at work when they are satisfied

Theme Alone and Together	
Subcategories	Categories
To have a diverse set of patients To practice good nursing To be wanted To not experience moral distress	The patient
To receive recognition To experience a supportive professional environment To experience fellowship	The co-worker
To feel safe To have appropriate responsibilities To self-manage To have and use competence	The registered nurse
To be given the precondition for self-management To have a diversity of tasks To have functional equipment To have an appropriate workload	The organisation

### Alone and together

The RNs in HHC experienced that they are both ‘alone’ and ‘together’. They are alone when dealing with the responsibility of care at the patients’ home, executing advanced care, assessing the patients’ needs in changing situations, and performing hidden tasks such as providing information to the next of kin. They are together with the patient concerning the challenges and resolutions; with their co-workers in discussing patients’ needs, challenges, and interventions, thereby experiencing recognition and a feeling of togetherness as both professionals and individuals; and the organisation in managing the patients’ needs within the provided framework.

### The patient

The RNs stated that they were satisfied with their work when the patients were diverse in age and caring needs. They believed that no other employment could provide this level of variation. The participants who had previously worked in other fields found a larger variety of patients in HHC, which contributed to their work satisfaction, stemming from diverse workdays would be diverse and that they were required to be able to consider and master several factors with diverse patients.

…if there’s anything I learned in my build-up education, it’s that you have to think about all types of conditions, you can’t focus solely on the diagnosis….

The RNs were satisfied when they delivered good quality nursing and felt that they were wanted by the patients. They also experienced stress regarding patient care when they felt that they were unable to meet the patient’s needs optimally, due either to their own performance or organisational issues.

…*waking up at night and thinking, oops, I forgot that or I should have spent a little more time with that patient. You can lie in bed and contemplate, because you have a bad conscience that you can’t leave at work*….

### The co-worker

The RNs stated that recognition from co-workers contributed to their work satisfaction. In the HHC, the RNs consider all the employees, not just other RNs, as co-workers. Some also viewed their leaders as co-workers. Recognition included praise from both first-level employees and management leaders. This also meant that colleagues listened to and respected the RNs’ professional assessments of patient situations.

*…it means a lot to me that I am respected. That is when I go to the manager or other employees and say what I think and mean and how the treatment should be in relation to the patient. I feel like they are listening to me, and that is very important to me*….

The results revealed that the work environment in HHC can be divided into two aspects affecting RNs’ work satisfaction: the professional environment and the fellowship. In the professional environment, discussions were centred on patients and their challenges were, with a focus on professional development and patient care. RNs could discuss challenges in patient care, receiving both guidance and support from co-workers. Experiences were shared where the RNs indicated that they would probably not be in the job if professional support from others was absent. Some only viewed other RNs as part of this environment, but others also viewed other healthcare workers and nursing assistants as part of this environment.

…wounds for example. One here has additional education in that and works a lot with it. I always ask her if she can say something about what she thinks if I’m unsure….

Like the professional environment, there was a experience of fellowship within the HHC. Spreading an uplifting mood and humour and being met with an uplifting mood are experienced by RNs when they are satisfied with their work in HHC. Contacting the co-worker when the RNs had finished their tasks was a common practice to ensure a more even spread of workload. The downside of this could be conflicts with other occupational groups; in particular, the RNs felt that they did not understand all the aspects of their work. Getting along with their colleagues in their private lives was also part of their work satisfaction, and the fact that they had succeeded in creating good relationships with others that extended beyond the work situation was experienced as valuable.

…it is important to have a good atmosphere and humour and that we have the time to talk together. Time for lunch, time for coffee. The morning coffee is very important; it is what makes me look forward to coming to work….

### The registered nurse

Being confident promoted work satisfaction, implying experiences of security linked to the RN’s abilities and prerequisites to handle various situations and work tasks. The prerequisites for competence and training are linked to the organisation, but in the RN’s experiences of work satisfaction, the sense of security belongs to the RNs themselves. The feeling of security and confidence was linked to work experience, and the RNs were familiar with the patient, the situation, or the procedure. The RNs experienced work satisfaction when they experienced an appropriate responsibility. The results revealed that experience of responsibility for the patient’s care could both contribute to and inhibit work satisfaction.

…I like the responsibility. To feel that responsibility in people’s homes. To feel that what I do is important and that it is up to me to solve it….

…I can feel like, my God, are we the ones who are going to take care of all this? … So, sometimes I feel a great responsibility that I just must sort this out somehow. There is no one else….

RNs in HHC experience work satisfaction when they are allowed to be independent and self-manage—in other words, when they were given autonomy to make decisions related to patient care and workload management within the given framework. They did not pay close attention to the time frames given, relying instead on their professional assessments of patient needs. Independence and self-management were foundational for their decision to work in HHC.

*‘…I do what I want, based on an assessment of the patient, of course. How I best meet patients’ needs is my decision. I should be mad if someone tries to override it. No, I do not bother with that…’* (Participant 7).

The RNs experienced work satisfaction when they had and used competence, which was considered important. While carrying out more advanced tasks was exciting, it should be supported with sufficient training to experience a feeling of mastery. The RNs also linked competency development to self-development and sometimes felt underappreciated.

…one must be made aware that it is actually a professional thing, just talking to people, when you ask the right questions….

### The organisation

The RNs experienced work satisfaction when they were provided with the prerequisites for self-management, making them feel trusted. These prerequisites provided by the organisation empowered them to manage their work and take the necessary measures independently without having to go through a system.

…I need to be allowed to make decisions, without having to go through some regulation or talk to anyone else. I just fix it, and then the system gets to trust my judgement….

In addition to patient diversity, the variation in work tasks contributed to their work satisfaction. The work tasks ranged from putting on support stockings to administering blood transfusions. The RNs found it exciting to have workdays filled with a variety of tasks and perceived exciting work as rewarding.

…I am most satisfied when there are varying tasks and varying working days. That I get to do different things….

The RNs reported that the equipment functionality influenced their work satisfaction. They stated that they had not been provided with sufficient clothing and that cars were not equipped for roads and weather conditions. This was perceived negatively by the RNs, because it was indicative of deprioritization and a lack of respect for their work. The RNs described missing or malfunctioning equipment in the patient’s homes as disrespectful and reflecting organizational oversight.

…I do not think they understand what I do. I can get quite frustrated when I get over there and the blood pressure device doesn’t work…it is what gets downgraded first, and it is the easiest to downgrade the equipment when things are as they are….

The RNs experienced work satisfaction when the workload was appropriate. The days when security alarms or other unplanned situations could be handled without affecting care quality were considered days with appropriate workloads. Although unforeseen events added excitement, they also induced stress if the workload was already high. The RNs described situations of appropriate workload as rewarding and positive but busy, providing a sense of focus.

…the days the programme is completely full and, of course, the alarm rings. Those days I feel like I can’t handle it anymore and the stress becomes totally overwhelming….

## Discussion

The results provide insights into RNs’ work satisfaction in municipal HHC. A key finding is that RNs derive work satisfaction from both independent and collaborative work settings—in other words working alone or together. In the HHC, the RNs often provide advanced care as the sole healthcare professional [27]. Consistent with previous research, these findings demonstrate that interpersonal interactions, including with both patients and co-workers, is a part of their work satisfaction [13–16, 21, 28]. If these interactions are supportive, they can prevent work-related stress and burnout [29, 30]. The present study underscores the importance of co-worker support in RNs’ work satisfaction, agreeing with a previous finding on co-worker support being crucial for retention [15]. However, RNs are missing opportunities to reflect upon ethical dilemmas and experience a lack of support from their managers [31]. Furthermore, RNs’ personal contacts may not be able to provide them with the needed support due to patient confidentiality limitations in HHC [32].

RNs in HHC experienced work satisfaction when they engaged in self-management practices, such as scheduling their own schedules, making assessments of patients’ needs, and prioritising their tasks. Autonomy has a known relationship with work satisfaction [13, 15, 16, 28] and is more important for RNs in HHC than those in institutional care [33]. The results of the present study further revealed that novice RNs had difficulties with the aforementioned self-management tasks. Not being able to provide good quality nursing influences satisfaction [34] and may lead to work-related distress [35]. First-line management in-home care consists of RNs who coordinate and lead the work group throughout the day [6]. RNs’ self-management may lead to RNs taking on too much, and in these cases, autonomy is linked to stress [15]. Self-management is a part of what RNs in HHC experience when they thrive. Thus, self-management may be difficult without work experience, which may affect work satisfaction. Further research is needed of RNs’ self-management in home healthcare.

Another factor influencing RNs’ work satisfaction was organizational support for self-management. When a case manager overrides RNs, work dissatisfaction may ensue [15]. By contrast, when RNs felt that their assessments were valued, respected, and validated by the case managers, they experienced work satisfaction.

The present study indicated that patient diversity (e.g. age, diagnosis, and situation) positively influenced RNs work satisfaction, in line with previous reports [21, 28]. Although patients in HHC tend to be older adults [2], they often present with a variety of ages, diagnosis, and specialised area [6]. Moreover, organisational and structural difficulties in providing competence adapted to patients’ care needs in HHC [36]. The present study also providesinsight that RNs without work experience of HHC may feel uncertain about evaluating patients’ needs and situations, underscoring the need for guidance and support to boost their confidence in their decision-making and self-management amidst patient diversity. Therefore, establishing formal mentorship programs is crucial to guide and support newly graduated RNs in HHC. The RNs also reported that they contacted more experienced co-workers, sometimes by contacting off-duty RNs informally, to discuss patients’ needs and situations and evaluate intervention strategies, particularly when RNs were the sole care provider for the patients. The phenomena ‘alone and together’ raised of this study merits further research.

### Limitations

To achieve trustworthiness, the analysis process was discussed with colleagues at several seminars [25]. To ensure the credibility of this study, RNs from different demographic areas who had experiences of HCC were included. This allowed to gather rich data. To increase the credibility, the analysis involved a critical and questioning approach by moving back and forth in the text. The intention was to clearly describe the method and results with descriptive quotations. A strength may be that the RNs worked in different work settings with different sizes in four municipalities. Although the sample size was small, the data obtained was rich and was sufficient to draw the necessary conclusions. However, the Norwegian context and the sample size limit the transferability of the findings to other populations. Nevertheless, the results are important and relevant to clinical practice. No ethical problems or conflicts occurred during the study.

### Implications for policy and practice

The results support that RNs derive work satisfaction from both independent and collaborative work settings—in other words working alone or together. This is particularly pertinent for managers in municipal HCC, whom have opportunity to facilitate and organize the framework for RNs work. The study also offers implications to consider in the structural organization of HHC, such as provide precondition for RNs’ self-management and ensure diversity of tasks.

## Conclusions

The present study identifies factors affecting RNs’ work satisfaction in municipal HHC: patients, co-workers, and organisation. Patients’ diversity positively influences RNs’ work satisfaction. Moreover, independent work and autonomy can also contribute to RNs’ work satisfaction in the municipal HHC. Awareness of these areas is essential for promoting RNs’ work satisfaction.

### Electronic supplementary material

Below is the link to the electronic supplementary material.

Supplementary Material 1

## Data Availability

The datasets used and analyzed during the current study are available from the corresponding author upon reasonable request.
