# Supplementary material for: Alone and together: registered nurses’ experiences of work satisfaction in municipal home healthcare
Source: BMC Nurs. 2024 Jun 5;23:382. doi: 10.1186/s12912-024-02051-3 (PMC11151470; doi:10.1186/s12912-024-02051-3)
Supplement: Supplementary file 1 — Supplementary Material 1 [file 12912_2024_2051_MOESM1_ESM.docx]

**Interview guide**

**_____________________________________________________________________**

*Question 1*. Can you tell me what it is like at work when you are most satisfied?

*Question 2*. Is there something that contributes to your work satisfaction that you wish to highlight?

*Follow-up questions* when necessary:

In what way?

Can you tell me more?

*Closing question*. Is there anything you want to add before ending the interview? **_____________________________________________________________________**
